# A feasible, low-cost, reproducible lamb’s head model for endoscopic sinus surgery training

**DOI:** 10.1371/journal.pone.0180273

**Published:** 2017-06-29

**Authors:** Henrique Fernandes de Oliveira, Valdes Roberto Bollela, Wilma Terezinha Anselmo-Lima, Carlos Augusto Pires de Oliveira Costa, Márcio Nakanishi

**Affiliations:** 1 Department of Otorhinolaryngology and Head and Neck Surgery, School of Medicine, Universidade de Brasília (UnB), Brasília, DF, Brazil; 2 Department of Infectious and Tropical Diseases, Ribeirão Preto School of Medicine (FMRP), University of São Paulo (USP), Ribeirão Preto, SP, Brazil; 3 Global Faculty of the Foundation for Advancement of Medical Education and Research (FAIMER), Philadelphia, PA, United States of America; 4 Department of Ophthalmology, Otorhinolaryngology and Head and Neck Surgery, FMRP, USP, Ribeirão Preto, SP, Brazil; 5 Department of Otorhinolaryngology and Head and Neck Surgery, School of Medicine, UnB, Brasília, DF, Brazil; Emory University School of Medicine, UNITED STATES

## Abstract

**Objective:**

To describe and standardize a reproducible, viable, low-cost lamb’s head model for endoscopic sinus surgery training.

**Methods:**

Otorhinolaryngology residents performed the following three endoscopic sinus surgeries using the lamb’s head model: inferior turbinectomy, bullectomy, and maxillary antrostomy. Each student dissected 10 specimens before training these procedures on human patients, and the benefit of the animal model training was evaluated.

**Results:**

Nineteen resident physicians of comparable academic level participated in training. All participants agreed that the lamb’s head model dissections improved their skills in using surgical and videoendoscopic instruments, 90% agreed that the dissections improved their confidence with respect to training in human patients, and all stated they would recommend the same training to future residents.

**Discussion:**

Lamb’s heads have been used for training in endoscopic sinus surgery. However, no standardization of this training had been performed to ensure that it is a valuable tool for learning and skill-building. The standardized method described in this study increased resident physicians’ skills and confidence before beginning their training on human patients. Moreover, our results demonstrate the feasibility of the model, considering its low cost and reproducibility.

**Conclusion:**

Future studies with this model should be conducted to assess whether the resulting increase in skills prevents and reduces medical errors, increases patient safety, reduces surgical costs, and improves the quality of otorhinolaryngological care.

## Introduction

Rhinology, particularly rhinologic surgery, has undergone major transformations in recent decades since the advent of videoendoscopy. The access routes of various nasal and paranasal surgical procedures have changed to incorporate the many benefits provided by the nasal endoscope, including smaller surgical scars, decreased operative time, and fewer complications.

However, this modality is a challenge for trainees and instructors. The nose is very near to the eyes, skull base, and oral cavity; an error during surgery or other iatrogenesis can cause permanent damage and may even be life-threatening [1, 2]. Therefore, accurate and extensive training of future otorhinolaryngologists during medical residency is essential.

The acquisition of surgical skills by trainees before performing procedures in human patients has become a requirement with respect to recommendations on the development of clinical practices that ensure patient safety in health care. Furthermore, such training reduces the duration of training on live patients, and training in realistic simulators can improve outcomes and reduce the odds of error [3].

Dissection of human cadavers, although ideal for training purposes, has become more expensive and strictly regulated on a global scale, due to ethical and legal issues [4]. Restrictions on the use of human heads for training and acquisition of surgical skills prompted the development of several alternatives in recent years, including synthetic models and virtual simulators; however, these models are costly and have limited availability [5–8].

Considering the difficulties of using cadavers and the limitations of models and simulators, a search for animal models suitable for training has become necessary.

The anatomy of nasal structures in the lamb is remarkably similar to that of humans. For this reason, the lamb’s head is an excellent surgical learning model and has been used successfully for otorhinolaryngology training in the past [9–11].

The use of a lamb’s head model enables the acquisition and refinement of skills required of resident physicians before using their skills in human patients. However, such training requires standardization and systematization so that the similarities of this model with human anatomy are highlighted and result in learning and acquisition of the skills required for training of otorhinolaryngologist surgeons.

The aim of this study was to describe and systematize a reproducible, feasible, and low-cost lamb’s head model for endoscopic sinus surgery training.

## Materials and methods

The study was approved by the relevant Institutional Animal Care and Use Committee at School of Medicine, Universidade de Brasília with protocol number 153674/2012. The research was approved in 2012 and data collection, and data collection took place from 2013 to 2016, which means that the research was conducted only after its approval. The above committee evaluates all investigations performed in the institution, including those with human subjects. Therefore, the committee approved the use of both humans and animals in the present study. All the residents that took part in this study gave written consent after they had received detailed information about the project. All procedures were conducted in exact compliance with the approved protocol.

Lamb’s heads were acquired from a regular, registered slaughterhouse in Núcleo Bandeirante, Brasília, DF, Brazil, at an approximate unit cost of USD3.00. Each specimen was provided clean and skinned and was kept frozen until use.

Approximately eight hours before dissection, the specimens were removed from the freezer. Once thoroughly thawed, the specimens were washed in running water to remove any clots and the outer surfaces were wrapped in ethanol-soaked gauze. Complete thawing is important to ensure that the texture of the nasal mucosa is similar to that of the analogous human structure. Moreover, the period of use should not exceed 4 hours to avoid decomposition of the specimen, with consequent loss of quality.

**Fig 1:**
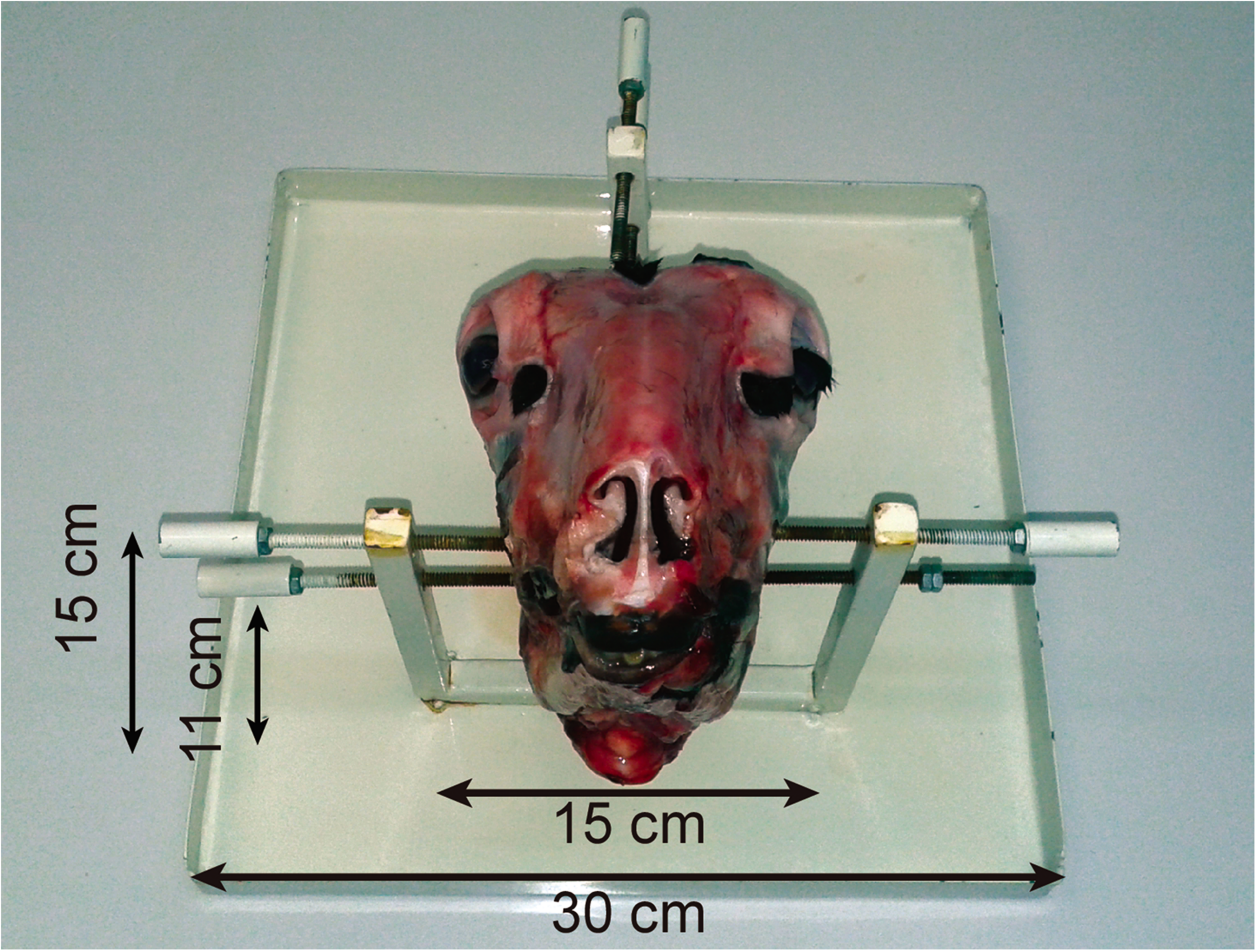
Lamb training support.

To facilitate handling, the lamb’s head was secured to a support fabricated specifically for this purpose. This support, designed with the aid of a machinist, was made from low-cost materials and consisted of small sheet-metal parts and three pairs of adjustable screws arranged in such a way as to allow fixation of the specimen (Fig 1). A commercial support developed by Karl Storz is also available.

A zero-degree rigid endoscope (HOPKINS®, Karl Storz, Neuhausen, Germany; diameter 4 mm, length 18 cm) was used. This endoscope was attached to a USB camera (Optice 2.0, Doctus Equipamentos Médicos, Brazil), a laptop, and a portable LED light source. Images of the training procedures were captured and stored on the laptop. The camera and light source were purchased together at an approximate cost of USD1,000.

The dissections were performed in the otorhinolaryngology laboratory by resident physicians under the supervision of a single researcher. Before training, each participant watched a demonstration video of a standard dissection performed by the researchers, and the steps and surgical objectives of training were clarified.

All resident physicians began training at the end of the second year of the residency program. This point in the program precedes endoscopic sinus surgery training in human patients, which is part of the third-year syllabus of our institution’s residency program.

**Fig 2:**
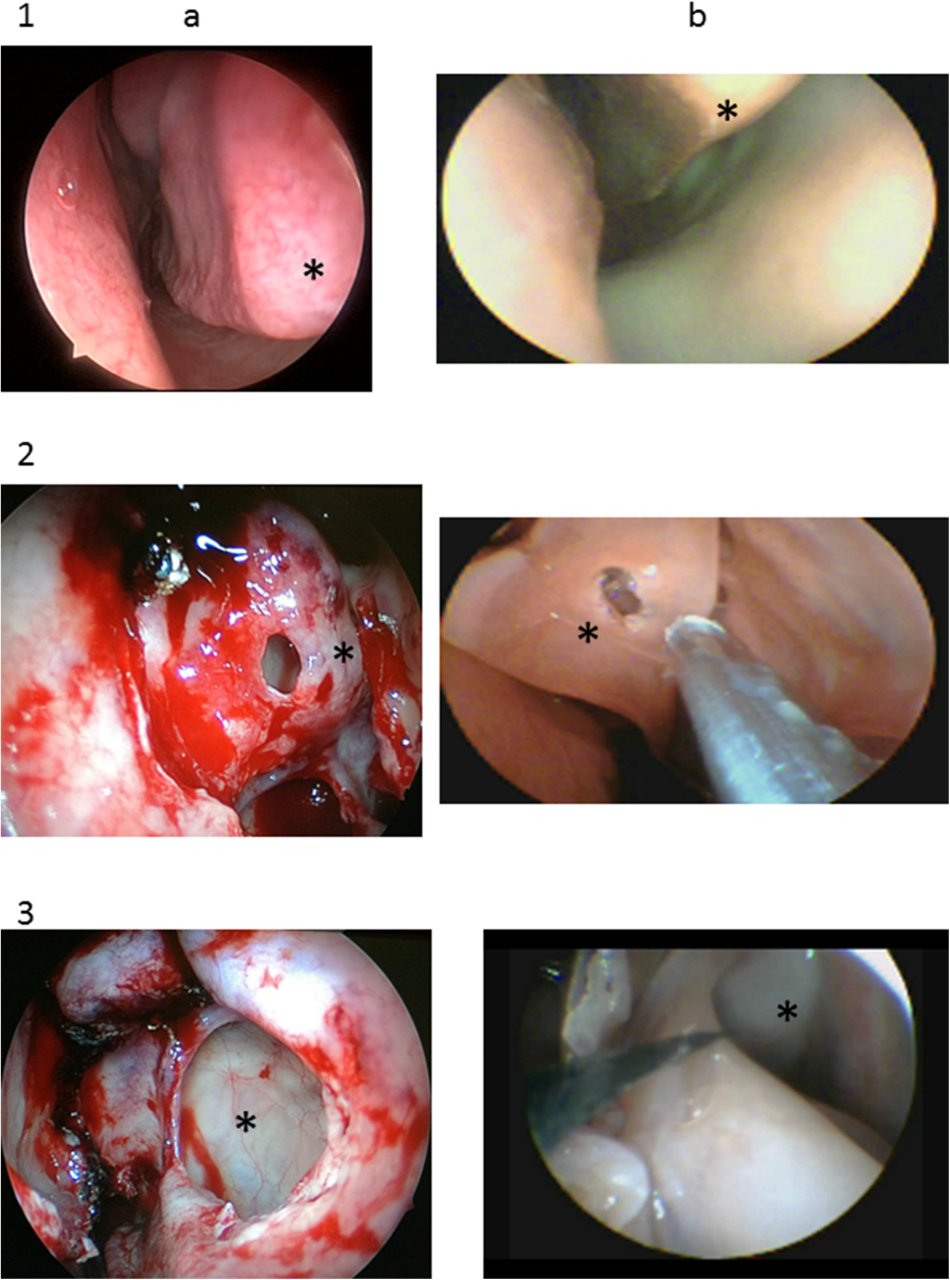
Human (a) and lamb (b) anatomy relevant to the three trained surgeries. 1. Inferior turbinate (*); 2. Bullectomy (*); 3. Maxillary sinus (*).

The researchers chose to train resident physicians in three procedures: inferior turbinate reduction, bullectomy, and maxillary sinus surgery. These procedures were selected because they are commonly performed in humans and are fully reproducible within the anatomical constraints of this animal model (Fig 2). Essentially, the difference is the longer turbinate in the lamb.

Inferior turbinate reduction was performed with the endoscope in the left hand and Heymann nasal scissors (Karl Storz). The proposed surgical model was a partial inferior turbinectomy of the head, body, and rear of the turbinate. Ethmoid bullectomy was performed after medial displacement of the middle turbinate, followed by use of through-cut, straight, Gruenwald-Henke nasal cutting forceps (Karl Storz) for complete removal of the anterior portion of the bulla. Maxillary antrostomy was performed after removal of the uncinate process with a sickle knife (Karl Storz), and the procedure was considered complete after exposure of the maxillary fontanelle.

All dissections were recorded using the image capture system of the camera installed on the computer. Each resident physician performed dissection 10 times, five in each nostril, in a total of 5 heads, always alternating sides, and always starting on the left side.

At the end of the dissections, the recorded videos were collected for later evaluation of the residents’ learning process. Several previously validated learning assessment tools could be used for this analysis.

Upon completion of the 10 dissections and after starting training of these procedures on human patients, the residents completed a questionnaire to rate their satisfaction and progress during training, as well as the personal perceptions of their skills, using a five-point Likert scale. The questionnaire is shown in Table 1.

**Table 1:** Training perceptions.

Dissections in the lamb model contributed to an increase in my skills using surgical and videoendoscopic instruments.	() Strongly agree
	() Agree
	() Neither agree nor disagree
	() Disagree
	() Strongly disagree
Dissections in the lamb model increased my confidence in performing surgery in human patients.	() Strongly agree
	() Agree
	() Neither agree nor disagree
	() Disagree
	() Strongly disagree
I would recommend lamb model dissections to resident otorhinolaryngologists before they begin training sinus surgery in human patients.	() Strongly agree
	() Agree
	() Neither agree nor disagree
	() Disagree
	() Strongly disagree

The completed questionnaires were then analyzed to formulate conclusions about residents’ expectations and assess their learning. The answers to each statement were converted to percentages and added.

## Results

A total of 19 residents (6 men and 13 women) from the same academic year and residency period performed the three surgeries proposed for each endoscopic dissection. Each resident performed 10 dissections in 5 lamb’s heads over an average period of 5 to 8 weeks. There were four cohorts of residents over 4 years.

A total of 190 videos were recorded in mp4 format. On average, each dissection lasted 33 minutes. The average duration was 31 minutes on the left side and 35 minutes on the right side.

Inferior turbinectomy, bullectomy, and maxillary antrostomy in the lamb’s head model were considered similar to the procedures performed in humans by 90%, 100%, and 95% of the residents, respectively.

All participants agreed that the dissections in the lamb model increased their skills with surgical and videoendoscopic instruments, 90% agreed that the dissections improved their confidence to begin training on human patients, and all stated they would recommend the same training to future residents.

## Discussion

Use of the lamb’s head model helped overcome the difficulties of using human cadavers and the limitations of alternative methods. This model complies with the principles of ethical animal use; because the study specimens do not have commercial value, this material is usually discarded after slaughter. Therefore, this training model is low-cost and complies with legal requirements.

The lamb’s head has been widely used in endoscopic sinus surgery, and its similarity to human anatomy has been well documented [9–11]. However, validation of the lamb’s head for surgical training is recent, and was established in a study by measuring the performance of different groups (medical students, residents with varying levels of expertise, and experienced surgeons) in a single dissection [12]. These findings validated the model for training purposes by proving that more experienced professionals performed better than beginners and students.

Similarities between our work and this previous study are the use of the lamb’s head and the use of video recording dissections. In fact, our study builds on the experience of Awad et al. (2015), but focusing only on the resident training scenario and on the use of the lamb’s head as an additional educational tool to offer safer environmental training for junior doctors.

Compared to the previous study, we sought to provide a thorough description of use of the established lamb’s head model for sinonasal surgery training. Detailed explanations of how the specimens were prepared, how the head support was fabricated, and of the tools and instruments employed were provided, as one of our intentions was to facilitate reproducibility anywhere with this low-cost, readily available model.

Another objective of our manuscript was to standardize training with the lamb’s head model specifically for surgical skill acquisition, focusing on individual skill-building and improvement; hence, each participant repeated the same step 10 times. In their validation of this model, Awad et al. did not measure individual learning because their sample was composed of distinct groups with very different levels of knowledge, and participants practiced only once on the model [12].

The primary objective of the standardization described in this study was to facilitate the acquisition of basic surgical skills and foster proper handling of surgical instruments. Dissection training using the animal model proposed herein will not teach residents how to treat a human disease; however, after having learned surgical skills during dissection, students will be better able to initiate training on humans under the supervision of an expert.

To avoid selection bias, we evaluated a sample composed exclusively of second-year residents, in contrast to other studies that evaluated resident physicians of different academic levels, senior otolaryngologists, fellows, and even undergraduate students [12–17]. For this reason, it took us 4 years to obtain a satisfactory sample size.

The lamb’s head model enabled the acquisition of skills using surgical and videoendoscopic instruments and improved the confidence of the resident physicians who were beginning endoscopic sinus surgery training, as demonstrated by the fact that all participants stated they would recommend the same training to future residents.

The proposed model is low-cost, requires little equipment, is portable, and can be replicated and used anywhere. Moreover, the recording of the dissections allows the evaluation of performance and learning at any time, thus obviating the need for supervision from teachers or advisors during the dissection itself; this increases trainee autonomy and saves valuable time.

Our study was innovative in that it standardized the training of resident physicians with the established lamb’s head model and explicitly described how to construct a low-cost version of this model.

Although human cadaver dissection is the best strategy to train for endoscopic sinus surgery, the high cost of obtaining these anatomical specimens and, particularly, limitations on its use owing to ethical and legal concerns have prompted a search for alternatives [4].

Several human head models have been developed from synthetic materials. These models have precise anatomical characteristics, and are portable and hygienic. However, their texture is poor compared with real human tissues [5, 6]. Another major disadvantage is their high cost and the need for disposal of the material after a single manipulation. Finally, some virtual simulators have been developed for surgical training [7, 8]. These models use advanced computer technology and are accurate in terms of anatomical measurements and reconstitution of three-dimensional images. However, they are not as effective as surgical manipulation of anatomical specimens or human patients.

The disadvantages of the lamb’s head model include the need for storage, the thawing period required before use, and the unavailability of lamb meat or slaughterhouses in some regions, although both are common in most countries.

The standardization proposed herein and the responses obtained demonstrate the relevance of using the lamb’s head model in the initial stages of surgical training, which will benefit patients. Furthermore, this study highlights the economic feasibility of the model, because the overall cost of both equipment and specimens is low. For these reasons, we recommend that the lamb’s head model be adopted for training of physicians who do not have access to human cadavers.

It is important to note that this study did not aim to compare resident physicians among one another but, rather, to compare the dissections made by each resident physician. Therefore, we assessed the progress of each trainee, respecting individual characteristics and skills.

However, further studies are necessary to evaluate the actual acquisition of skills by residents using the lamb’s head model. This can be achieved by assessing surgical learning using tools previously validated for this purpose [18].

## Conclusions

Nasal dissection using the lamb’s head model proposed in this study is a viable, low-cost alternative to the use of human cadavers. Future studies with this model would help demonstrate whether the resulting acquisition of skills can prevent and reduce medical errors, increase patient safety, decrease surgical costs, and improve the quality of otorhinolaryngological care.

## References

[1] Kinsella JB, Calhoun KH, Bradfield JJ, Hokanson JA, Bailey BJ. Complications of Endoscopic Sinus Surgery in a Residency Training Program. Laryngoscope. 1995;105(10):1029–32. doi: 10.1288/00005537-199504000-00007 7564829 10.1288/00005537-199510000-00003

[2] Hosemann W, Draf C. Danger Points, Complications and Medico-Legal Aspects in Endoscopic Sinus Surgery. GMS Curr Top Otorhinolaryngol Head Neck Surg. 2013;12:Doc06 doi: 10.3205/cto000098 24403974 10.3205/cto000098 PMC3884541

[3] World Health Organization (WHO). WHO Patient Safety Curriculum Guide for Medical Schools [Internet]. World Health Organization (WHO) 2009 Available from: http://apps.who.int/iris/bitstream/10665/44091/1/9789241598316_eng.pdf.

[4] Zuckerman JD, Wise SK, Rogers GA, Senior BA, Schlosser RJ, DelGaudio JM. The Utility of Cadaver Dissection in Endoscopic Sinus Surgery Training Courses. Am J Rhinol Allergy. 2009;23(2):218–24. doi: 10.2500/ajra.2009.23.3297 19401053 10.2500/ajra.2009.23.3297

[5] Briner HR, Simmen D, Jones N, Manestar D, Manestar M, Lang A, et al Evaluation of an Anatomic Model of the Paranasal Sinuses for Endonasal Surgical Training. Rhinology. 2007;45(1):20–3. 17432064

[6] Nogueira JF, Stamm AC, Lyra M, Balieiro FO, Leao FS. Building a Real Endoscopic Sinus and Skull-Base Surgery Simulator. Otolaryngol Head Neck Surg. 2008;139(5):727–8. doi: 10.1016/j.otohns.2008.07.017 18984272 10.1016/j.otohns.2008.07.017

[7] Arora H, Uribe J, Ralph W, Zeltsan M, Cuellar H, Gallagher A, et al Assessment of Construct Validity of the Endoscopic Sinus Surgery Simulator. Arch Otolaryngol Head Neck Surg. 2005;131(3):217–21. doi: 10.1001/archotol.131.3.217 15781761 10.1001/archotol.131.3.217

[8] Fried MP, Sadoughi B, Gibber MJ, Jacobs JB, Lebowitz RA, Ross DA, et al From Virtual Reality to the Operating Room: The Endoscopic Sinus Surgery Simulator Experiment. Otolaryngol Head Neck Surg. 2010;142(2):202–7. doi: 10.1016/j.otohns.2009.11.023 20115975 10.1016/j.otohns.2009.11.023

[9] Gardiner Q, Oluwole M, Tan L, White PS. An Animal Model for Training in Endoscopic Nasal and Sinus Surgery. J Laryngol Otol. 1996;110(5):425–8. 8762308 10.1017/s0022215100133882

[10] Mladina R. Endoscopic Surgical Anatomy of the Lamb's Head. Tuttlingen, Germany: Endo-Press; 2014.

[11] Mladina R, Vukovic K, Stern Padovan R, Skitarelic N. An Animal Model for Endoscopic Endonasal Surgery and Dacryocystorhinostomy Training: Uses and Limitations of the Lamb's Head. J Laryngol Otol. 2011;125(7):696–700. doi: 10.1017/S0022215111000776 21693073 10.1017/S0022215111000776

[12] Awad Z, Taghi A, Sethukumar P, Tolley NS. Construct Validity of the Ovine Model in Endoscopic Sinus Surgery Training. Laryngoscope. 2015;125(3):539–43. doi: 10.1002/lary.24927 25200556 10.1002/lary.24927

[13] Touska P, Awad Z, Tolley NS. Suitability of the Ovine Model for Simulation Training in Rhinology. Laryngoscope. 2013;123(7):1598–601. doi: 10.1002/lary.23974 23361620 10.1002/lary.23974

[14] Mladina R, Skitarelic N. Training Model for Endoscopic Sinus Surgery. Am J Rhinol Allergy. 2013;27(3):251 doi: 10.2500/ajra.2013.27.3886 23710963 10.2500/ajra.2013.27.3886

[15] Mladina R, Castelnuovo P, Locatelli D, Duric Vukovic K, Skitarelic N. Training Cerebrospinal Fluid Leak Repair with Nasoseptal Flap on the Lamb's Head. ORL J Otorhinolaryngol Relat Spec. 2013;75(1):32–6. doi: 10.1159/000347080 23548498 10.1159/000347080

[16] Awad Z, Arora A, Touska P, Tolley NS, Darzi A. Validating the Sheep Model in Rhinology Skills Training. Otolaryngol Head Neck Surg. 2012;147(2 suppl):P119 doi: 10.1177/0194599812451438a263

[17] Delgado-Vargas B, Romero-Salazar AL, Reyes Burneo PM, Vasquez Hincapie C, de Los Santos Granado G, Del Castillo Lopez R, et al Evaluation of Resident's Training for Endoscopic Sinus Surgery Using a Sheep's Head. Eur Arch Otorhinolaryngol. 2016;273(8):2085–9. doi: 10.1007/s00405-015-3877-1 26739302 10.1007/s00405-015-3877-1

[18] Martin JA, Regehr G, Reznick R, MacRae H, Murnaghan J, Hutchison C, et al Objective Structured Assessment of Technical Skill (OSATS) for Surgical Residents. Br J Surg. 1997;84(2):273–8. 9052454 10.1046/j.1365-2168.1997.02502.x

